# Gestational Diabetes Mellitus and Risk Factors in a Multi‐Ethnic National Case–Control Study

**DOI:** 10.1002/edm2.70005

**Published:** 2024-10-07

**Authors:** Barbara M. Daly, Zhenqiang Wu, Lynne Chepulis, Robert K. R. Scragg

**Affiliations:** ^1^ School of Nursing, Faculty of Medical and Health Sciences University of Auckland Auckland New Zealand; ^2^ Department of Geriatric Medicine University of Auckland Auckland New Zealand; ^3^ Waikato Medical Research Centre, School of Health University of Waikato Hamilton New Zealand; ^4^ School of Population Health University of Auckland Auckland New Zealand

**Keywords:** ethnicity, gestational diabetes, risk factors, smoking

## Abstract

**Introduction:**

Gestational diabetes mellitus (GDM) continues to increase particularly for non‐European women. This study aimed to identify and quantify risk factors for women diagnosed with gestational diabetes in New Zealand to identify women at higher risk.

**Methods:**

A national dataset of 601,166 eligible women who had ≥ 1 birth in New Zealand between January 2001 and December 2010 identified 11,459 women with gestational diabetes of whom 11,447 were randomly matched with 57,235 control women for age and year of delivery.

**Results:**

Adjusted odds ratios (95% CI) showed higher odds of gestational diabetes for Asian (3.60, 3.39–3.82), Pacific (2.76, 2.57–2.96) and Māori (1.23, 1.15–1.31) women compared with European/Other women. Women most economically disadvantaged (1.44, 1.34–1.56), not registered with a lead maternity carer (1.16, 1.04–1.30) and those identified as smokers (1.20, 1.11–1.31) were more likely than control women to develop gestational diabetes. In contrast, women residing in rural (0.83, 0.77–0.88) and remote areas (0.68, 0.60–0.77) were less likely to develop gestational diabetes compared with women living in urban areas, and similarly for non‐New Zealand resident women (0.78, 0.72–0.85) compared with resident women.

**Conclusions:**

Women who were diagnosed with gestational diabetes were more likely to be non‐European, economically disadvantaged, residing in urban areas, unregistered with a lead maternity carer and more likely to smoke. In addition to universal screening for pre‐existing diabetes, all women at risk of gestational diabetes should be identified and supported to undertake to a 75 g glucose challenge test between 24 and 28 weeks.

## Graphical abstract

Women at greatest risk of developing GDM are Asian, Pacific and Māori women, and those who are more economically deprived and smoke. Improving nutritional intake, physical activity, weight gain and smoking cessation may reduce the risk of developing GDM.

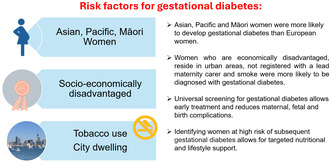

**Summary**

- What is already known?
  ○ Risk factors for gestational diabetes have been described, but it is unclear if they are the same in a multi‐ethnic New Zealand population.
- What this study has found?
  ○ Women at greatest risk of gestational diabetes were Asian, Pacific and Māori women, women living in greater deprivation and women who smoked. The proportion of Māori women diagnosed with gestational diabetes is lower than expected suggesting a lack of screening.
- What are the implications of the study?
  ○ In addition to universal screening in early pregnancy for diabetes, it is important to identify women at high risk of subsequent gestational diabetes for targeted nutritional and lifestyle support, and for screening for gestational diabetes between 24 and 28 weeks gestation.

## 1 Introduction

Gestational diabetes mellitus (GDM) continues to increase in New Zealand (NZ), from 1.3% in 2001 [1] to 6.2% in 2019 [2]. The obesity epidemic is the greatest driver of GDM [3, 4], with a linear increase from < 10% of women with a body mass index (BMI) < 19 kg/m [2] developing GDM to over 30% for women with a BMI > 40 kg/m^2^ [5]. Demographic changes, European and Asian women delaying childbirth and increased fertility rates are also major contributors of GDM with Pacific (2.19) and Māori (2.07) women having higher fertility rates compared with all women (1.75) in 2018 and 1.64 in 2021 [6].

Regional reports show there are large variations in the prevalence of GDM among different ethnic groups in NZ. Approximately 13% of national births occur in National Women's hospital in Auckland where Indian women have the highest prevalence (25%), followed by Pacific and North Eastern Asian women (19%), Middle Eastern, Latin American and African women (17%), Māori women (8%) and European women (6%) [5]. Similar ethnic‐specific prevalences have been reported for the Waikato region, being significantly increased for older Asian, Māori and Pacific women but not European women [7]. The prevalence is likely under‐reported for Māori women due to lower screening rates [1, 5, 8, 9], and fewer Māori women are offered a fasting glucose test that is more sensitive in detecting elevating serum glucose levels for Māori women than an HbA_1c_ or a 2‐h oral glucose tolerance test (OGTT) [9]. Higher diagnostic thresholds in NZ (≥ 5.5 and ≥ 9.0 mmol/L) [1] for fasting glucose and 2‐h OGTT, respectively, compared with those recommended internationally (≥ 5.1 and ≥ 8.5 mmol/L) [1], likely increase the ethnic variation [9, 10]. GDM also varies by region with the highest prevalence in Auckland (8.2%) and lowest in the Wairarapa in the lower North Island (1.4%) [1].

Established major risk factors for developing GDM include a prepregnancy BMI ≥ 22 kg/m^2^ in Asian populations [1, 3] and BMI ≥ 25 kg/m^2^ in European populations [3], early pregnancy weight gain [11], parity, increasing maternal age [3], non‐European ethnicity [11], genotype [11], familial history of GDM [11], previous pre‐eclampsia, preterm delivery [3], hypertension [11], polycystic ovarian disease [11] and cigarette smoking [12]. Universal HbA1c screening identifies women with existing diabetes for treatment, and facilitates targeted nutritional and weight gain advice for women with elevated Hba1c levels [1]. Women with an elevated HbA1c and treated before 24 weeks (median 13 weeks) were less likely to develop pre‐eclampsia or deliver prior to 37 weeks or have infants > 90th centile for bodyweight compared with women who underwent a 75 g OGTT between 24 and 28 weeks and treated after 24 weeks (median 28 weeks), suggesting early treatment is beneficial [13]. Although major risk factors are well documented within different populations, there is a paucity of reports for national multi‐ethnic populations.

The aims for this study are to identify and quantify sociodemographic risk factors associated with the development of GDM in a large national, multi‐ethnic population.

## 2 Methods

A matched case–control design was used, utilising a large maternal retrospective national database that included all women who had at least one birth in New Zealand between January 2001 and December 2010 and described previously [14]. Ethical approval was not required for this study as all identifying data were encrypted and anonymised datasets utilised. This was confirmed by the NZ Health and Disability Ethics committee (20 January 2023).

### 2.1 Ministry of Health National Datasets

**FIGURE 1:**
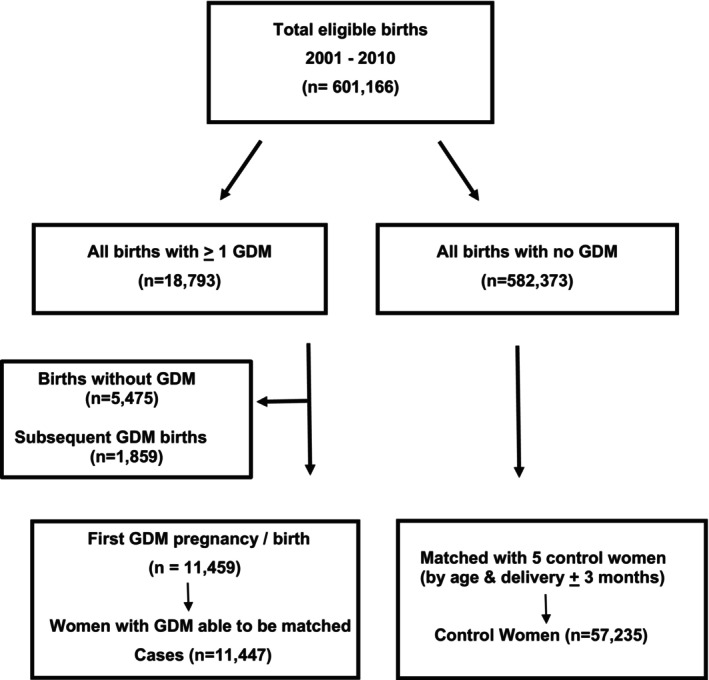
Total births in New Zealand from January 2001 to December 2010 identified 11,447 women with gestational diabetes mellitus who were randomly matched with 57,235 control women without diabetes by age and year of birth.

Utilising the National Maternity Collection (MAT) dataset, a total of 604,398 births were identified between January 2001 and December 2010 in NZ. The MAT dataset collects biographical information from primary maternity services and lead maternity providers and services. After excluding mothers with missing data for gender (*n* = 264), those aged < 15 years or ≥ 50 years (*n* = 728) and women identified with existing diabetes prior to pregnancy (*n* = 2240), resulted in 601,166 births. Women identified with GDM were associated with 18,793 births, and women not identified with any diabetes had a total of 582,373 births (Figure 1).

To exclude women with Type 1 or 2 diabetes, the National Pharmaceutical Collection Data Mart (PHARMS) were used to identify women dispensed hyperglycaemic‐related medications prior to, and during and after pregnancy—utilising the method to construct the NZ virtual diabetes register [14]. The National Minimum Dataset (NMDS) provided hospital‐based information during pregnancy, birth and the postnatal period.

### 2.2 Study Population

Of the total of 18,793 births for women identified with GDM in the maternal dataset, 5475 births not associated with GDM and an additional 1859 GDM‐associated births were excluded, to avoid duplication of women diagnosed with GDM. Of the 11,459 women diagnosed with GDM, 11,447 were each able to be randomly matched for age and year of delivery (±3‐months) with five control women without diabetes (*n* = 57,235) from a total of 582,373 births (Figure 1).

### 2.3 Screening for GDM 2000–2010

Screening and diagnosis of GDM for women during this period were based on identifying women with risk factors for GDM and recommending an early OGTT. For lower risk women, and depending on which centre they were attending, it was recommended they have a nonfasting 50 g glucose challenge screen (polycose) or a 75 g OGTT between 24 and 28 weeks [15]. An OGTT was recommended for women who had a 1‐h serum glucose ≥ 7.8 mmol/L following the polycose test [15] and diagnostic of GDM if > 11.0 mmol/L. [5] A diagnosis of GDM was confirmed following a 75 g OGTT if fasting glucose was ≥ 5.5 mmol/L at 1 ‐h or ≥ 9 mmol/L at 2 ‐h. Under diagnosis of GDM was expected during this period as approximately 20%–89% of pregnant women had a polycose test that varied by District Health Board [15] that was less sensitive than the 75 g 2‐h OGTT, and a proportion of women with serum glucose ≥ 7.8 mmol/L did not have a follow up 75 g OGTT test [16].

### 2.4 Statistical Analysis

SAS 9.4 (SAS Institute, Cary, NC, 2013) was used for all statistical analysis. Frequency and percentages were generated using PROC FREQ. Adjusted odds ratios (OR, 95% CI) were calculated using logistic regression. The following covariates were included in the regression model: age, year of delivery, social deprivation status (based on residential addresses), smoking at registration or smoking and/or tobacco counselling at delivery or 2 weeks postdelivery and cardiovascular dispensed medications (mostly antihypertensives). Information for maternal BMI was not available in the MAT dataset and could not be adjusted for in the multivariate analyses.

## 3 Results

Of the 11,459 women identified during their first GDM‐associated pregnancy, 11,447 women were randomly matched with 57,235 (control) women from a total of 582,373 births for women without diabetes (Figure 1).

Asian and Pacific women were over represented as cases. Asian women made up 24% of the cases compared with 10% of the controls, Pacific women (19% compared with 9%), Māori women (15% compared with 18%) and European/Other women made up 42% of cases compared with 64% of control women (Table 1).

**TABLE 1:** Multivariate odds ratios (OR) for factors associated with women identified with gestational diabetes mellitus (GDM, *n* = 11,447) compared with women without GDM (*n* = 57,235) matched for age and year of delivery.

Variable and level	GDM *n*, (%)	No GDM *n*, (%)	OR (95% CI)	*p* ^a^	OR (95% CI) adjusted	Adjusted *p* ^b^
Ethnicity						
European/Other	4757 (42)	36,431 (64)	1.00		1.00	
Asian	2743 (24)	5651 (10)	3.74 (3.54–3.95)	< 0.0001	3.60 (3.39–3.82)	< 0.0001
Pacific	2200 (19)	5123 (9)	3.31 (3.12–3.51)	< 0.0001	2.76 (2.57–2.96)	< 0.0001
Māori	1747 (15)	10,030 (18)	1.35 (1.27–1.43)	< 0.0001	1.23 (1.15–1.31)	< 0.0001
Deprivation status^c^						
Highest	1384 (12)	9856 (17)	1.00		1.00	
High	1611 (14)	10,413 (18)	1.11 (1.02–1.19)	0.01	1.06 (0.98–1.15)	0.16
Middle	2160 (19)	11,077 (20)	1.40 (1.30–1.51)	< 0.0001	1.29 (1.19–1.39)	< 0.0001
Low	2587 (23)	11,751 (21)	1.59 (1.48–1.71)	< 0.0001	1.36 (1.27–1.47)	< 0.0001
Lowest	3685 (32)	13,757 (24)	1.94 (1.81–2.08)	< 0.0001	1.44 (1.34–1.56)	< 0.0001
Missing data	20	375				
Region health authorities						
Northern	6093 (53)	23,262 (41)	1.00		1.00	
Mid North Island	1874 (16)	10,323 (18)	0.69 (0.65–0.73)	< 0.0001	1.04 (0.97–1.10)	0.29
Lower North Island	1565 (14)	11,422 (20)	0.52 (0.49–0.56)	< 0.0001	0.67 (0.63–0.72)	< 0.0001
Southern	1904 (17)	12,081 (21)	0.60 (0.57–0.64)	< 0.0001	0.96 (0.90–1.02)	0.17
Missing data	11	147				
Urban/Rural status						
City	8592 (78)	37,446 (68)	1.00		1.00	
Semi‐rural	707 (6)	4918 (9)	0.63 (0.58–0.68)	< 0.0001	0.95 (0.88–1.04)	0.29
Rural	1371 (13)	10,012 (18)	0.60 (0.56–0.63)	< 0.0001	0.83 (0.77–0.88)	< 0.0001
Remote	319 (3)	2743 (5)	0.51 (0.45–0.57)	< 0.0001	0.68 (0.60–0.77)	< 0.0001
Missing data	458	2116				
Resident status						
Yes	10,558 (92)	53,476 (94)	1.00		1.00	
No	887 (8)	3369 (6)	1.34 (1.24–1.44)	< 0.0001	0.78 (0.72–0.85)	< 0.0001
Registered maternity lead						
Yes	10,618 (93)	54,263 (95)	1.00		1.00	
No	829 (7)	2972 (5)	1.43 (1.32–1.55)	< 0.0001	1.16 (1.04–1.30)	0.01
No of previous births^**d**^						
Nil	4023 (40)	19,032 (37)	1.00		1.00	
1	2870 (29)	16,703 (32)	0.81 (0.77–0.86)	< 0.0001	0.83 (0.78–0.87)	< 0.0001
2–3	2147 (22)	12,274 (24)	0.83 (0.78–0.88)	< 0.0001	0.81 (0.76–0.86)	< 0.0001
4–6	809 (8)	3287 (6)	1.16 (1.07–1.27)	0.0006	0.95 (0.86–1.05)	0.31
> 7	124 (1)	515 (1)	1.14 (0.93–1.39)	0.22	0.89 (0.72–1.10)	0.28
Missing data	1474	5424				
Pre‐eclampsia						
No	10,100 (88)	54,387 (95)	1.00		1.00	
Yes	1347 (12)	2848 (5)	2.55 (2.38–2.70)	< 0.0001	2.29 (2.13–2.46)	< 0.0001
Smoking postdelivery^e^						
No	4015 (35)	21,457 (38)	1.00		1.00	
Unknown	6172 (54)	29,076 (51)	1.35 (1.26–1.44)	< 0.0001	1.18 (1.10–1.27)	< 0.0001
Yes	1260 (11)	6702 (12)	1.12 (1.04–1.21)	0.004	1.20 (1.11–1.31)	< 0.0001
CV medications pre‐delivery^f^						
No	10,900 (95)	56,214 (98)	1.00		1.00	
Yes	547 (5)	1021 (2)	2.78 (2.50–3.10)	< 0.0001	2.18 (1.95–2.45)	< 0.0001

^a^ Adjusted for age and year of delivery.

^b^ Adjusted for all variables as tabulated.

^c^ Deprivation status (highest 1–2, high 3–4, middle 5–6, low 7–8 and lowest 9–10).

^d^ Controlling for maternal age when matching explains the unexpected OR for GDM by number of births (Appendix 1).

^e^ Smoking at registration or smoking and/or tobacco counselling at delivery or 2 weeks postdelivery.

^f^ Cardiovascular (CV) dispensed drugs (antihypertensives, diuretics, anticoagulants, cardiac, nitrates and lipid lowering).

Table 1 also outlines univariate (after matching for age and delivery period) and multivariate OR after adjusting for all covariates, including ethnicity, deprivation status, region of residency and rural or urban based, NZ residency, registration with a lead maternity carer, number of previous births, pre‐eclampsia, smoking status and dispensed cardiovascular (mostly hypertensive) medications prior to delivery. Multivariate (OR, 95% CI) showed significantly higher odds of GDM for Asian (3.60, 3.39–3.82), Pacific (2.76, 2.57–2.96) and Māori (1.23, 1.15–1.31) women compared with European/Other women. Women who were most economically disadvantaged (1.44, 1.34–1.56), not registered with a lead maternity carer (1.16, 1.04–1.30) and women identified as smokers at registration or during delivery or 2 weeks postdelivery (1.20, 1.11–1.31) were more likely to develop GDM compared with control women. Women who were diagnosed with GDM were more likely to develop pre‐eclampsia (2.29, 2.13–2.46) and be prescribed cardiovascular medications (mostly antihypertensives) prior to delivery (2.18, 1.95–2.45). In contrast, women had a lower risk of developing GDM: if residing in remote (0.68, 0.60–0.77) and rural (0.83, 0.77–0.88) areas compared with urban‐based women; if residing in the lower North Island (0.67, 0.63–0.72) compared with women living in the Northern region; women without permanent residency (0.78, 0.72–0.85) compared with NZ resident women. Results for GDM by number of births contradict the expected increase in GDM because women with GDM were matched by age with control women. Maternal age is a confounder for number of births and GDM. Univariate analyses for the total cohort (*n* = 601,166) show that GDM increases by number of births as expected (Appendix 1).

## 4 Discussion

After adjusting for all confounding variables, Asian women were almost four times, and Pacific women almost three times, more likely to develop GDM compared with European women. Māori women were approximately 25% more likely than European women to develop GDM, and a similar proportion of Māori women was identified as cases (15%) and controls (18%) supporting other reports of underdiagnosis [5, 8]. The lower than expected prevalence for Māori women is likely due to lower screening rates for GDM [7, 8, 9], fewer undergo a fasting glucose screening test [9] (i.e., more sensitive than an OGTT for Pacific women and possibly Māori women) and women with a higher pre‐ or early pregnancy BMI [10]. The higher diagnostic thresholds in NZ for the 2‐h OGTT (≥ 9 mmol/L) and fasting glucose (≥ 5.5 mmol/L) [5] than those recommended (≥ 5.3 mmol/L), given the linear association between increasing birthweight and adverse outcomes, likely exacerbate the underdiagnosis for Māori women [1, 13]. The prevalence of GDM for Indian NZ women is higher than for women in India [17] and higher for Asian women who have immigrated to Australia and New Zealand [18]. The same trend is seen in Middle Eastern and North African women [19], compared with women in NZ who have immigrated from those continents, and possibly a consequence of living in an environment that leads to an increase in BMI [20], and/or reflects different screening patterns.

Women were more likely to develop GDM if they were not registered with a lead maternity carer, or economically disadvantaged as previously reported in New Zealand [2] and North America [21], with an evident dose–response effect for economic deprivation (Table 1). Women diagnosed with GDM were more likely to develop pre‐eclampsia and be prescribed cardiovascular (mostly hypertensive) medications prior to delivery. A similar association has also been reported for women diagnosed with GDM in their second pregnancy, after developing hypertension or pre‐eclampsia during their first pregnancy [3]. Women identified as smokers were more likely to develop GDM than women who did not smoke. The association between smoking and GDM has been previously reported in a 2021 meta‐analysis; [12] however, it may be due to heterogeneity between studies, as a more recent meta‐analysis did not find a significant association [22].

In contrast, women who were non‐NZ residents, residing in the lower North Island, and in rural or remote areas, were less likely to develop GDM compared with resident women and those living in the Northern region and in urban areas, respectively. Possibly nonresident women who have spent less time in NZ are more protected from developing GDM [20]. Regional differences in the prevalence of GDM have been reported previously in NZ [2].

The association between maternal age and increasing number of pregnancies and developing GDM is well documented [3]. Matching women with GDM with control women by maternal age explains the unexpected results for primipara compared with primigravida women in the multivariate analysis (Table 1). Univariate OR for the total cohort of 601,166 women show the expected increase in GDM by number of births (Appendix 1). BMI could not be controlled for in the multivariate analyses and typically increases with maternal age and each pregnancy [3]. Women already insulin resistant are additionally challenged during pregnancy, when placental hormones further increase insulin resistance to facilitate glucose availability to the foetus, and/or beta cells are unable to sufficiently upregulate and secrete sufficient insulin, to maintain euglycaemia [3, 23].

The 2022 National Women's Health report that 70% of Asian women during pregnancy have a BMI < 25 kg/m^2^ and only 7% > 35 kg/m^2^ is comparable with European women where 60% have a BMI < 25 kg/m^2^ but have the lowest prevalence of GDM [5]. Although BMI is most strongly associated with GDM, early screening for a high BMI and other risk factors [11] allows early pregnancy nutritional and weight gain counselling, and timely screening for GDM. A recent Indian study reporting on 600 women who attended an antenatal care clinic in the first trimester found that 10% developed GDM within the first 12 weeks of pregnancy [17], although they may have had pre‐existing dysglycaemia.

In addition to pregnancy‐related complications affecting women who develop GDM, up to 45% remain glucose intolerant at 6–16 weeks postpartum [1], yet postpartum screening is low [1, 24, 25]. Women who develop GDM have a high risk of developing cardiometabolic conditions following delivery [26]. Developing GDM also increases the risk of epigenetic changes in the foetus potentially leading to increased body weight in childhood and cardiometabolic conditions in adulthood [27]. Reducing the risk of developing GDM remains a challenge. Lifestyle intervention trials show a reduced risk of developing GDM for at‐risk women [23], through increasing physical activity [23, 28, 29], and nutritional interventions [29].

It is expected that these results and reports [23, 24, 28, 29] will lead to improved guideline recommendations for screening in pregnancy and increased funding to ensure all women, especially Māori women, are screened for pre‐existing diabetes and GDM. Ideally, women at risk of GDM are identified early in pregnancy and counselled to improve their nutritional intake and lifestyles as a first line strategy, to reduce weight gain and to ensure they have an early second trimester 75 g OGTT.

### 4.1 Strengths and Limitations of the Study

A major limitation for this study was not being able to control for BMI as that is not recorded in the MAT dataset. Under or overestimated OR may have occurred for variables with significant proportions of missing data. For example, the positive association between smoking and women developing GDM may have been overestimated if more control women smoked (with missing data) than women diagnosed with GDM. Despite these limitations, this is a large national study that included all pregnant women giving birth in all NZ hospitals over a 10‐year period and quantifies risk factors for an ethnically diverse group of women who developed GDM.

## 5 Conclusion

Findings in this large national study show that women at greatest risk of developing GDM were Asian, Pacific and Māori women, more economically deprived and women who smoke. Identifying and targeting modifiable risk factors including nutritional intake, physical activity, gestational weight gain and smoking may potentially reduce the development of GDM in high‐risk populations. Furthermore, programmes designed to optimise screening of GDM in these group would likely support improved maternal and foetal outcomes.

## Author Contributions

All authors have approved of the final version for publication. Barbara M. Daly and Robert K.R. Scragg conceptualised and designed the study and interpreted the results. Barbara M. Daly acquisitioned funding and datasets, conducted aspects of the data analysis, interpreted the results and wrote the manuscript. Zhenqiang Wu curated the datasets, analysed the data, interpreted the results and contributed to writing the manuscript and the revised version. Robert K.R. Scragg and Lynne Chepulis critically appraised the study findings and reviewed and evaluated the manuscript. Barbara M. Daly, Zhenqiang Wu and Robert K.R. Scragg had full access to the data, are responsible for the integrity of the data and the accuracy of the data analysis.

## Ethics Statement

Encrypted and anonymised datasets were used for this study. Because it was not possible to identify women, ethical approval was not required, and this was confirmed by the NZ Health and Disability Ethics committee (20 January 2023).

## Conflicts of Interest

The authors declare no conflicts of interest.

## Data Availability

Aggregated data are available on request.

## Appendix 1

### A.1 Maternal age, prevalence of gestational diabetes mellitus (GDM) and univariate odd ratios (OR) by number of pregnancies or births for the total cohort (*n* = 601,166)

**Table:**

No. of previous births/pregnancies	Number of previous births			No. of previous pregnancies OR (95% CI)
	Maternal age, *N*; mean (SD)	GDM, *n* (%)	No GDM, *n* (%)	
0	219,205; 27.1 (6.3)	4060 (1.85)	215,145 (98.15)	1.00
1	174,021; 29.6 (5.7)	3578 (2.06)	170,443 (97.94)	1.11 (1.06–1.16)
2–3	117,028; 31.2 (5.4)	2680 (2.29)	114,348 (97.71)	1.24 (1.18–1.31)
4–6	28,352; 33.2 (5.0)	1017 (3.59)	27,335 (96.41)	1.97 (1.84–2.11)
> 7	3665; 36.4 (4.3)	169 (4.61)	3496 (95.39)	2.57 (2.19–3.00)
Missing data		1814 (3.08)	57,018 (96.9)	
